# The Effect of Noseband Tightening on Horses’ Behavior, Eye Temperature, and Cardiac Responses

**DOI:** 10.1371/journal.pone.0154179

**Published:** 2016-05-03

**Authors:** Kate Fenner, Samuel Yoon, Peter White, Melissa Starling, Paul McGreevy

**Affiliations:** 1 Kandoo Equine, Towrang, New South Wales, Australia; 2 Faculty of Veterinary Science, The University of Sydney, Camperdown, Sydney, New South Wales, Australia; University of Minnesota, UNITED STATES

## Abstract

Restrictive nosebands are common in equestrian sport. This is concerning, as recent evidence suggests that very tight nosebands can cause a physiological stress response, and may compromise welfare. The objective of the current study was to investigate relationships that noseband tightness has with oral behavior and with physiological changes that indicate a stress response, such as increases in eye temperature (measured with infrared thermography) and heart rate and decreases in heart rate variability (HRV). Horses (*n* = 12) wearing a double bridle and crank noseband, as is common in dressage at elite levels, were randomly assigned to four treatments: unfastened noseband (UN), conventional area under noseband (CAUN) with two fingers of space available under the noseband, half conventional area under noseband (HCAUN) with one finger of space under the noseband, and no area under the noseband (NAUN). During the tightest treatment (NAUN), horse heart rate increased (*P* = 0.003), HRV decreased (*P* < 0.001), and eye temperature increased (*P* = 0.011) compared with baseline readings, indicating a physiological stress response. The behavioral results suggest some effects from bits alone but the chief findings are the physiological readings that reflect responses to the nosebands at their tightest. Chewing decreased during the HCAUN (*P* < 0.001) and NAUN (*P <* 0.001) treatments. Yawning rates were negligible in all treatments. Similarly, licking was eliminated by the NAUN treatment. Following the removal of the noseband and double bridle during the recovery session, yawning (*P* = 0.015), swallowing (*P* = 0.003), and licking (*P* < 0.001) significantly increased compared with baseline, indicating a post-inhibitory rebound response. This suggests a rise in motivation to perform these behaviors and implies that their inhibition may place horses in a state of deprivation. It is evident that a very tight noseband can cause physiological stress responses and inhibit the expression of oral behaviors.

## Introduction

Nosebands have long been a popular piece of equestrian equipment. The simplest form is the cavesson noseband, traditionally loosely fitted and unrestrictive. 'Crank' (sometimes called ‘cranked’) nosebands, developed in the 1980s [1] and frequently used in equestrian sports today, are similar to a plain cavesson noseband with the addition of a leveraged buckle to allow for tighter fit. Their purpose can best be understood through the standards upheld by the Fédération Equestre Internationale (FEI), which is the international governing body of equestrian sports. The FEI stipulates that horses should demonstrate submissiveness, and penalises “resistance, evasion, putting out the tongue, or teeth grinding” [2]. 'Submission' is a term used in the discipline of dressage to describe a horse’s attentiveness, willingness and confidence to behave with lightness and ease in the way it executes different movements [2]. Therefore, submission “does not mean subordination, but an obedience revealing its presence by a constant attention and a state of relaxation” [2]. It is therefore beneficial in competition for horses to appear submissive, and nosebands may be used to give this appearance.

It is thought that, chiefly by pressing the bit(s) against the tongue, a tight noseband restricts tongue movements, which are among the mechanisms by which horses attempt to dissipate pressure from the bit within the oral cavity [3]. The resultant inability to escape bit pressure leads to the sensitisation of the horse’s mouth, increasing the horse’s responsiveness to rein pressure [4] and thereby making the horse appear more responsive [5]. The contemporary crank noseband can be tightened to the extent that it can compromise vascular perfusion [6], and may even cause nerve and bone damage [7]. The use of these crank nosebands and the extreme tightening of nosebands is thought to be increasing in equestrian sports [6], and is likely to have an impact on the welfare of the horse, but this impact has not yet been quantified.

The aim of this study was to investigate the influence that increasing noseband tightness has on the behavior and physiology of horses. Observations of oral and non-oral behaviors were recorded concurrently with measures of physiological function, specifically heart rate and heart rate variability (HRV) and eye temperature. An infrared thermographic camera was used to measure eye temperature. These data were collected at four different levels of noseband tightness to quantify the effect of noseband tightness on horse behavior and stress responses. An increase in heart rate, a decrease in HRV, and an increase in eye temperature indicate a physiological stress response. The study also sought to identify any ‘post-inhibitory rebound’ in behaviors, which is the term given to an increase in the expression of a behavior from baseline following a period of restriction. A post-inhibitory rebound is thought to represent a negative welfare state during the period of inhibition, as it indicates a build-up of motivation [8]. The welfare consequences of preventing behaviors that exhibit post-inhibitory rebound are considered more profound than those of preventing behaviors that do not [9]. Post-inhibitory rebound in horses after removal of a restrictive noseband would manifest as a higher frequency of a behavior than before the noseband was fitted.

## Materials and Methods

The protocol and conduct of this study were approved by the University of Sydney Animal Ethics Committee, New South Wales, Australia (AEC protocol number 2013/5967).

### Horses

Twelve horses of various ages (mean 6.6 ± 3.6 years), breeds (one Warmblood, four Australian Stock Horses, two Clydesdale-crosses, one Thoroughbred, one Percheron, two Andalusians and one Appaloosa), sex (two mares, three stallions, and seven geldings), and height (mean 155.4 ± 7.2 cm) were recruited into the study. All horses had been started under saddle, with various levels of subsequent training experience. Some had worn a snaffle bridle with a loose cavesson noseband previously (*n* = 4). However, prior to the experiment none of the test horses had ever worn a double bridle (a bridle with two bits, as is required at higher levels in competitive dressage) or crank noseband. Naïve horses were deliberately selected in order to explore the impact of the devices on horses that have not habituated to them. Habituation is likely to introduce unquantifiable variability into responses as different horses are likely to habituate at different rates and to different extents. The horses used were housed in four paddocks (approximately 10 acres/ 4.1 ha), with a mixture of the mares and four geldings in one paddock, and three stallions and three geldings in another, all at Kandoo Equine, Towrang, Australia. All horses were in good condition with no signs of injuries, sickness, or disease. Each horse had free access to water and improved pasture as well as supplementation with a mixture of lucerne or clover hay. All horses were handled on a daily basis for feeding and health-check purposes.

### Treatments

Each horse was randomly allocated to one of the four treatment groups per day, undergoing each level of noseband tightness over four consecutive days. Over a three-week period (end of July to August 2015), four horses were tested per day (Monday to Thursday). The horses were brought in individually from the pasture wearing a standard webbing headcollar and cotton lead rope, and were led to the preparation area.

The four treatments were assigned to horses using a predetermined randomised order that had been generated in Microsoft Excel (Microsoft Corporation, One Microsoft Way, Redmond, WA) and spread out across test days. Sampling occurred at the same time each day, between 9:30 and 14:00 h, to reduce any effects of circadian rhythms. The same operator carried out all treatments and was blinded to the treatment status of the horse until the second phase of the experiment when the treatment was applied. Each horse was first allowed to stand for two minutes for acclimatization purposes. After this two-minute period, the horse was groomed to ensure that the head, neck, and girth area were clean and uninjured in any way. A heart rate monitor girth was then fitted to each horse, with a video camera capturing the starting point of the heart rate monitoring period. The horse was then bridled with either a cob- or a full-sized standard double bridle that comprised a curb bit, a bridoon (which is a small single jointed snaffle bit) and curb chain. Each bridle was fitted with a padded crank noseband that was left unfastened until the horse entered the test area. Once the horse was prepared for the treatment, it was then brought inside the stable barn and led into the test area. The test area comprised a custom-built bay constructed from hay bales that were covered with soft canvas material. This bay was 3 m x 2 m x 1m in size and prevented the horse from turning around while being tested, and thus turning their head away from the thermal imaging and video cameras. Once the horse was in the test bay, the ten-minute baseline session began. Readings of eye temperature were taken each minute, behavior was video recorded continuously, and heart rate recordings were obtained every second.

Once baseline readings were collected, each horse then underwent their assigned treatment. Treatment sessions of ten minutes commenced after the baseline session. Treatments consisted of fitting the crank noseband, using the taper gauge developed by the International Society for Equitation Science [10] to one of the following conditions:

1. unfastened noseband (UN),
2. conventional area under noseband (CAUN, fastened to a conventional degree, allowing two fingers to fit in the space under the noseband as measured by a taper gauge that allows a standard analogue of the circumference of two adult fingers [9.89 ± 0.21 cm] under the noseband in the nasal midline [6]),
3. half conventional area under noseband (HCAUN, as measured by a taper gauge that allows a standard analogue of one adult finger under the noseband in the nasal midline, rather than the conventional two adult fingers [6]) and,
4. no area under noseband (NAUN, that allowed no part of the standard taper gauge under the noseband in the nasal midline).

After each treatment, horses were allowed to stand in the bay without the bridle for ten-minutes, designated as the recovery period. During this period, a single rail wooden gate at chest level restrained the horse. Eye temperature, heart rate and HRV, and behavioral observations were again recorded during this recovery period to collect data on post-inhibitory responses. After the 10-minute baseline reading, a ten-minute treatment period, and a ten-minute recovery period (total of 30 minutes), the horse was led out of the test bay, and held outside in the preparation area where the heart rate monitor and video recorder were turned off and removed to coincide with the completion of the treatment. Once the treatment had finished and all equipment had been removed, the horse was led back to its original paddock. A familiar handler then led the next horse to the preparation area for the subsequent randomly allocated treatment, repeating the above preparation protocol.

### Heart rate and heart rate variability

Horses were fitted with a Polar Equine Electrode Set–H2 Wearlink Transmitter and girth enabling the attachment of a Polar RX800CX Heart Rate Monitor (Polar Electro Oy, Professorintie 5, 90440 Kempele, Finland) for continuous recording of heart rate and HRV, measured as inter-beat intervals. The girth was fitted firmly around the horse’s thorax to the left-hand side girth region on the horse, the area having previously been clipped to assist in recording of cardiac activity. Clipping was necessary as the experiment was conducted in the winter months, and all of the horses had grown a dense hair-coat, which is known to interfere with the heart rate sensor electrode. The heart rate monitor was fitted immediately before horses were led into the test bay, and removed once the horse had concluded the sampling period and returned to the preparation area. Ultrasound transmission gel (Lectron II Conductivity Gel, New Jersey, USA) was applied liberally to the heart rate electrode girth to optimise the electrical contact to the tissue and to maximise the accuracy of readings. At the end of each sampling period, the stored heart rate and HRV data were downloaded onto a computer for storage and later analysis.

### Infrared thermography

**Fig 1:**
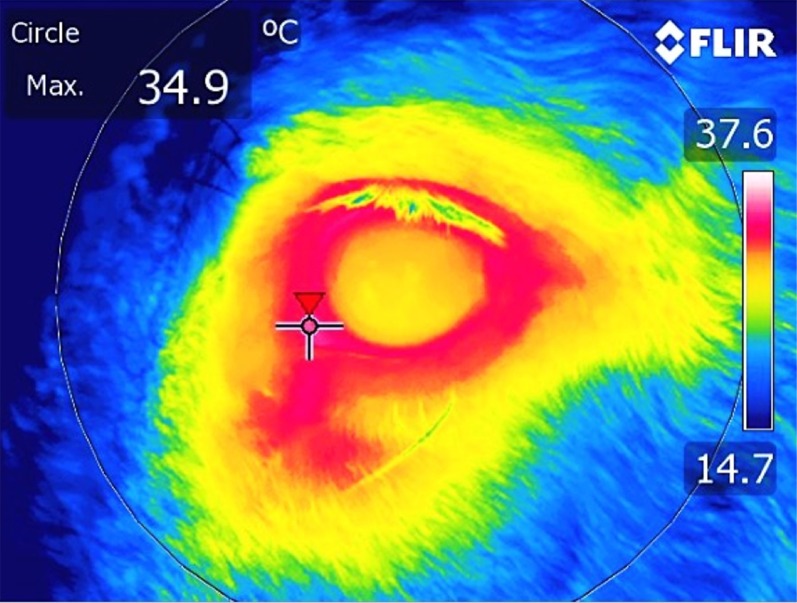
An example of an infrared image of the eye region. The cross indicates the position of the maximum temperature within the area of the eye used for analysis, the medial posterior palpebral border of the lower eyelid and the lacrimal caruncle.

An infrared camera (ThermaCam T604, FLIR Systems AB, Danderyd, Sweden) was used to collect thermographic images of each horse’s left eye. This camera has a thermal sensitivity of <0.02°C, and can detect temperature over a range of − 20°C to 250°C. Once thermal images were collected, they were uploaded to FLIR Tools and Research IR® thermal analysis software. Analysis of thermal imaging was undertaken to determine minimum and maximum eye temperature. The maximum temperatures (°C) within the area of the medial posterior palpebral border of the lower eyelid and the lacrimal caruncle (Fig 1) were recorded every minute throughout the entire sampling period, as in the method described by Yarnell [11]. All horses were scanned from the same side (left), from a distance of one metre. Ambient temperature and relative humidity were recorded at 30 minute intervals inside the stable barn, and these values were entered into the camera’s settings to allow for atmospheric changes during the sampling period, as previously recommended by Stewart [12].

### Behavior and software

Each horse was videoed using a Sony HDR-PJ790 camera (Sony, Australia) and Sony Handycam DCR-SR62 (Sony, Australia) on tripods, one in front and one on the right-hand side of the horse. The cameras recorded physical and vocal activity during each treatment. Video scoring was then performed using the observational software *Noldus The Observer XT version 11*.*5* (Noldus IT 206/354 Eastern Valley Way, Chatswood). All 11 observed behaviors (Table 1) were scored over the entire 30-minute sampling period for each animal.

**Table 1:** Coding criteria of horse behaviors observed and recorded in the Observer program.

Behavior (Horse)	Description
LICKING	Tongue extends out of the buccal cavity. Tongue may move across the muzzle
CHEWING	The horse bites and softly grinds the teeth.
YAWNING	The horse separates the maxilla and mandible and opens the buccal cavity. The tongue extends somewhat out of the buccal cavity
SWALLOWING	Cranio-caudal movement of the throat, specifically the gullet
BLINKING	Closing movement of the eyelids.
HEAD SHAKING	The horse shifts the head from side to side
HEAD TOSS	The horse lifts the head above shoulder height
HEAD NOD	The horse lowers and raises the head more than once
CRIB-BITE	The horse bites a horizontal object with the incisors while arching the neck
WOOD CHEW	The horse chews with the incisors on the wooden barrier
EAR MOVEMENTS	Ears forward: The ears are observed to turn rostrally
	Ears lateral: The ears are rotated laterally and dorsal/caudally. The opening of the inner ear is observed to turn outwards
	Ears back: The ears are flattened (abducted)

The beginning and end of each observation was also scored in *The Observer*, allowing the heart rate and HRV data to be aligned with behavioral records.

### Statistics

Eye temperature, heart rate and heart rate variability were analysed using a split-plot randomised block analysis of variance (ANOVA) with horses as blocks, treatment as the whole-plot treatment and session as the split-treatment. A multivariate REML was used to obtain the correlations among the three variates. There were 12 treatment combinations in this experiment and we were focusing on only half of the possible pair-wise comparisons because these directly address the research questions. We are not declaring differences to be significant or not at a 5% level, rather presenting the actual P value for each difference and allowing the reader to agree or disagree with the conclusions we draw.

The behavior data were all counts and were analysed using a generalised linear mixed model with a Poisson distribution, a logarithm link function and the same error structure as the split-plot ANOVA. Statistical analysis of data was undertaken in GenstatTM 17th edition (VSN International Ltd, Waterhouse Street, Hemel Hempstead, UK).

## Results

### Heart rate and heart rate variability

**Fig 2:**
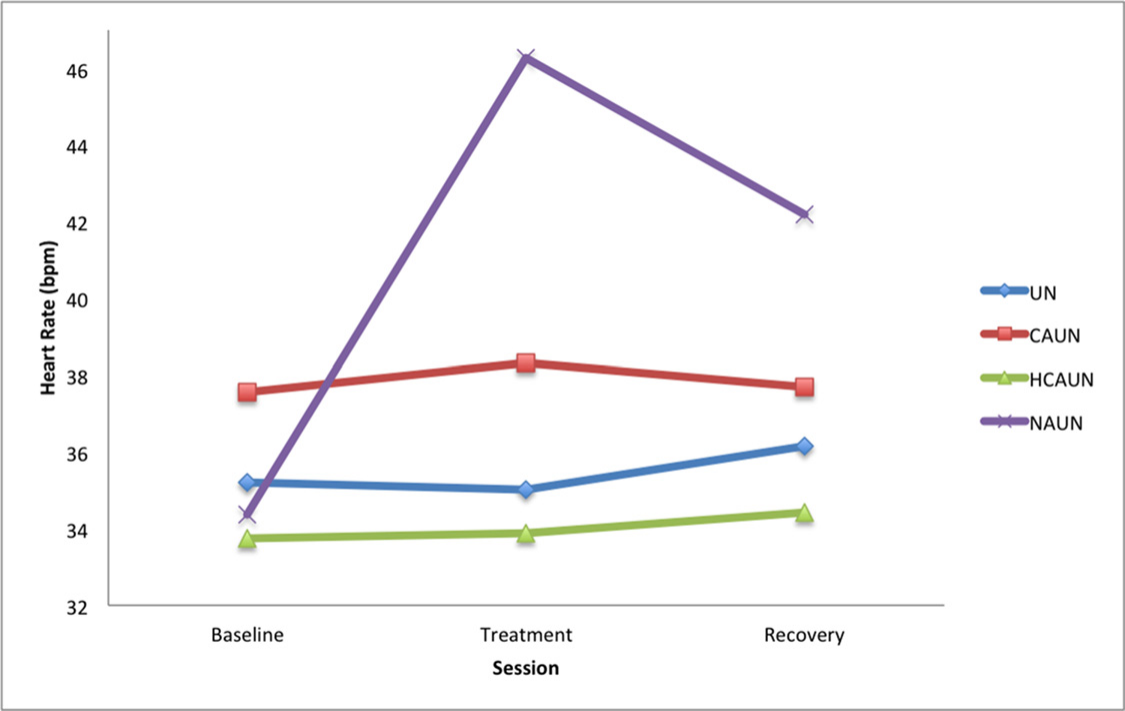
Mean heart rates in horses (beats per minute, *n* = 12) when wearing a double bridle and an unfastened crank noseband (UN), a conventionally fastened noseband (CAUN), a noseband fastened with half the conventional area underneath (HCAUN), and noseband fastened with no area underneath (NAUN).

**Fig 3:**
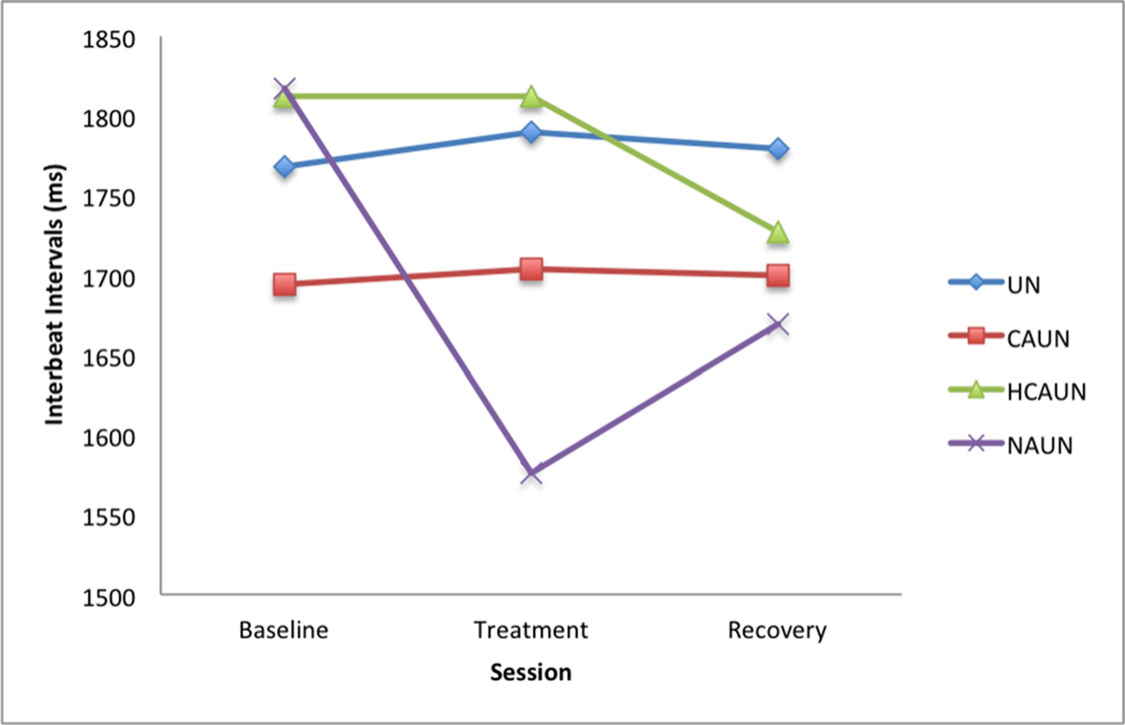
Mean heart rate variability in horses (*n* = 12) in response to wearing a double bridle and an unfastened crank noseband (UN), a conventionally fastened noseband (CAUN), a noseband fastened with half the conventional area underneath (HCAUN), and noseband fastened with no area underneath (NAUN).

Heart rate correlated with HRV (corr = −0.744, *P* < 0.001), as expected. However, the correlations between eye temperature and heart rate (corr = −0.143, *P* = 0.744) and eye temperature and HRV (corr = 0.083, *P* = 0.514) when sampled each minute were not significant. There was a significant increase in heart rate for the NAUN treatment (*P* = 0.003) during treatment and recovery sessions compared with the baseline session (see Fig 2 and Table 2). This was accompanied by a significant decrease in HRV for the NAUN treatment (*P* < 0.001) between baseline and the treatment (see Fig 3 and Table 3).

**Table 2:** Heart Rate.

	Treatment			
Session	UN	CAUN	HCAUN	NAUN
	Means			
Baseline	35.21	37.56	33.74	34.37
Treatment	35.01	38.33	33.89	46.28
Recovery	36.16	37.69	34.42	42.19
	Differences			
Treatment—Baseline	-0.2	0.8	0.1	11.9
Treatment—Recovery	-1.1	0.6	-0.5	4.1
	*P* values			
Treatment—Baseline	0.961	0.844	0.970	**0.003**
Treatment—Recovery	0.771	0.871	0.893	0.300

*P* values for comparisons of heart rate (beats per minute) among the four treatments, with averaged means (s.e.d. = 3.92; d.f. = 88) in horses (*n* = 12) in response to wearing a double bridle and an unfastened crank noseband (UN), a conventionally fastened noseband (CAUN), a noseband fastened with half the conventional area underneath (HCAUN), and noseband fastened with no area underneath (NAUN).

**Table 3:** Heart Rate Variability.

	Treatment			
Session	UN	CAUN	HCAUN	NAUN
	Means			
Baseline	1768	1694	1812	1817
Treatment	1790	1704	1812	1576
Recovery	1779	1700	1727	1669
	Differences			
Treatment—Baseline	21.6	9.5	0.2	-241.1
Treatment—Recovery	11.1	4.0	84.9	-92.9
	*P* values			
Treatment—Baseline	0.761	0.894	0.998	**0.001**
Treatment—Recovery	0.876	0.956	0.235	0.193

*P* values for comparisons of heart rate variability among the 4 treatments, with averaged means (s.e.d. = 70.90; d.f. = 88) in horses (*n* = 12) in response to wearing a double bridle and an unfastened crank noseband (UN), a conventionally fastened noseband (CAUN), a noseband fastened with half the conventional area underneath (HCAUN), and noseband fastened with no area underneath (NAUN).

### Eye temperature

**Fig 4:**
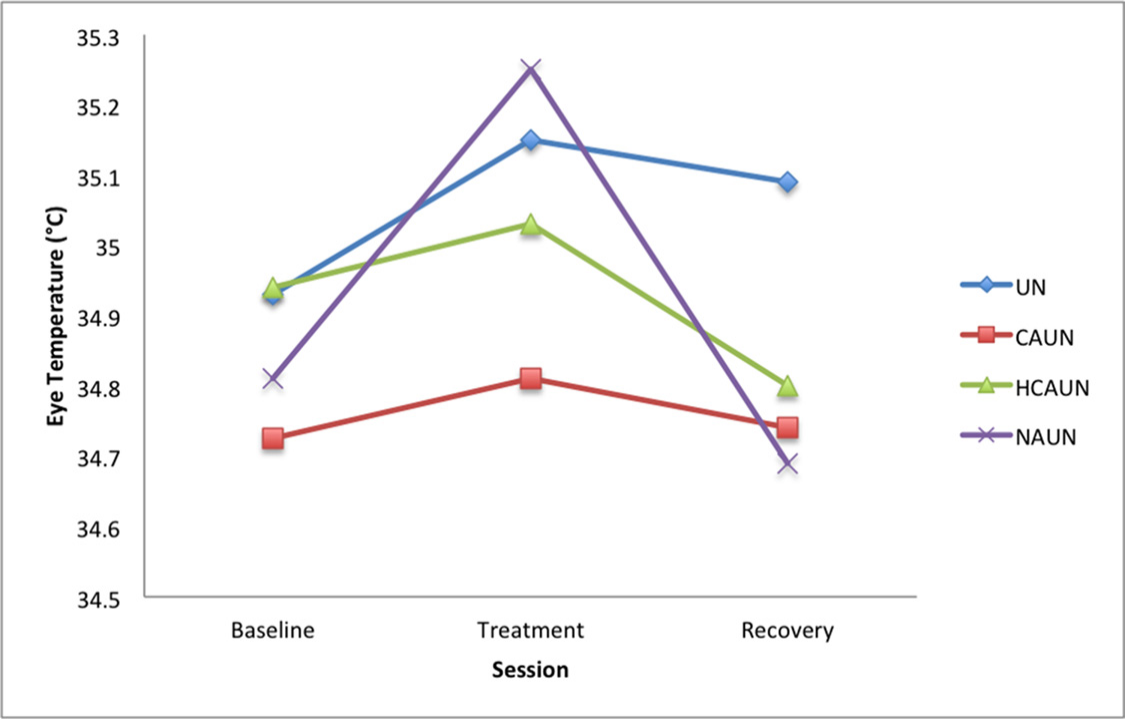
Mean eye temperatures horses (in degrees Celsius; *n* = 12) in response to wearing a double bridle and an unfastened crank noseband (UN), a conventionally fastened noseband (CAUN), a noseband fastened with half the conventional area underneath (HCAUN), and noseband fastened with no area underneath (NAUN).

Eye temperature increased between baseline and treatment sessions in the NAUN (*P* = 0.011) treatment. Eye temperature then decreased in the NAUN recovery period (*P* < 0.001) (see Fig 4, Table 4).

**Table 4:** Eye Temperature.

	Treatment			
Session	UN	CAUN	HCAUN	NAUN
	Means			
Baseline	34.93	34.73	34.94	34.81
Treatment	35.15	34.81	35.03	35.25
Recovery	35.09	34.74	34.80	34.69
	Differences			
Treatment—Baseline	0.22	0.08	0.10	0.44
Treatment—Recovery	0.06	0.06	0.24	0.56
	*P* values			
Treatment—Baseline	0.202	0.301	0.567	**0.011**
Treatment—Recovery	0.714	0.703	0.168	**0.001**

*P* values for comparisons of eye temperature (in degrees Celsius) among the four treatments, with averaged means (s.e.d. = 0.1697; d.f. = 88) in horses (*n* = 12) in response to wearing a double bridle and an unfastened crank noseband (UN), a conventionally fastened noseband (CAUN), a noseband fastened with half the conventional area underneath (HCAUN), and noseband fastened with no area underneath (NAUN).

### Behavior

**Fig 5:**
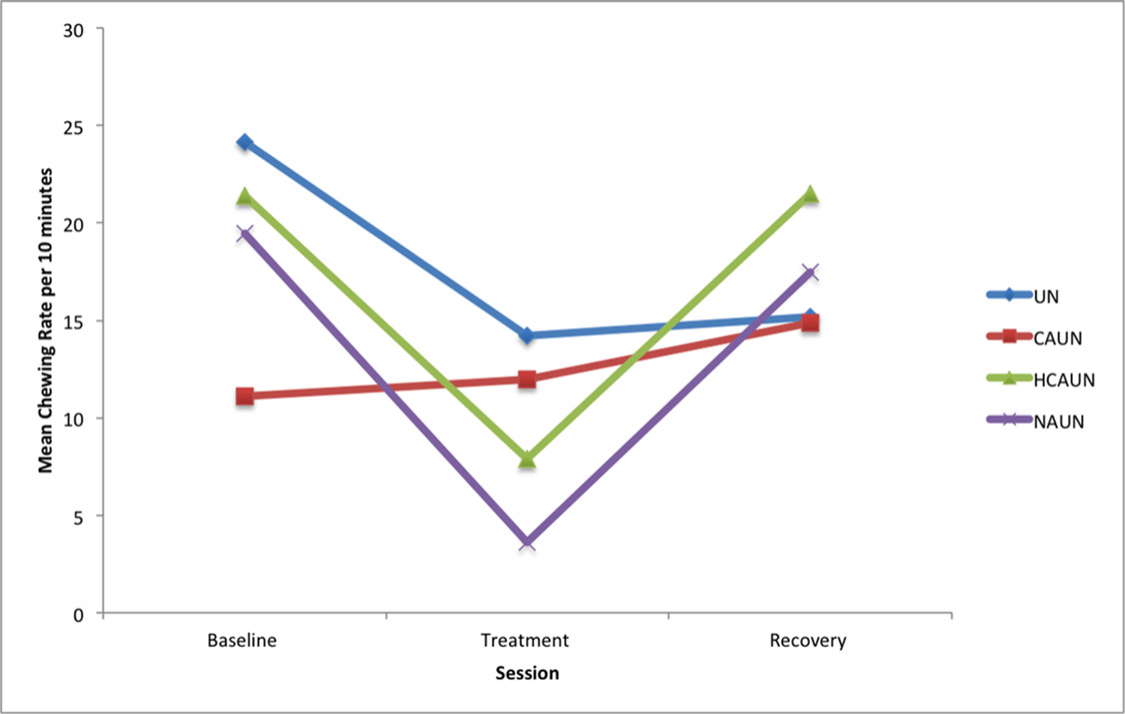
Mean chewing rates per ten-minute session in horses (*n* = 12) in response to wearing a double bridle and an unfastened crank noseband (UN), a conventionally fastened noseband (CAUN), a noseband fastened with half the conventional area underneath (HCAUN), and noseband fastened with no area underneath (NAUN).

Horses exhibited significantly less chewing in the treatment session compared to baseline readings in treatments HCAUN (*P* < 0.001) and NAUN (*P* < 0.001). This was followed by a significant increase in chewing during the recovery session compared to the treatment session for both HCAUN (*P* < 0.001) and the NAUN (*P* < 0.001; see Fig 5, Table 5), indicating that these treatments inhibited chewing. After the UN treatment, there was significantly less chewing than in the preceding 20 minutes (p<0.03).

**Table 5:** Chewing.

	Treatment			
Session	UN	CAUN	HCAUN	NAUN
	Back-transformed means			
Baseline	24.14	11.09	21.41	19.47
Treatment	14.20	11.99	7.88	3.61
Recovery	15.18	14.86	21.50	17.50
	ratio (s.e.d on log-ratio scale)			
Treatment / Baseline	0.59 (0.21)	1.08 (0.27)	0.37 (0.27)	0.19 (0.37)
Treatment / Recovery	0.94 (0.24)	0.81 (0.25)	0.37 (0.27)	0.21 (0.37)
Recovery / Baseline	0.63 (0.21)	1.34 (0.25)	1.00 (0.20)	0.9 (0.21)
	P value			
Treatment / Baseline	**0.016**	0.774	**< 0.001**	**< 0.001**
Treatment / Recovery	0.794	0.398	**< 0.001**	**< 0.001**
Recovery / Baseline	**0.030**	0.252	1.000	0.619

*P* values for comparisons of chewing among the four treatments, with back transformed means and ratios in horses (*n* = 12) in response to wearing a double bridle and an unfastened crank noseband (UN), a conventionally fastened noseband (CAUN), a noseband fastened with half the conventional area underneath (HCAUN), and noseband fastened with no area underneath (NAUN).

**Fig 6:**
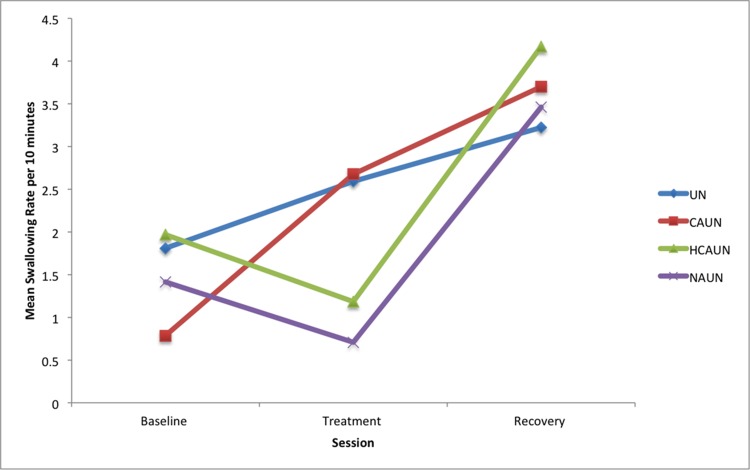
Mean swallowing rates per ten-minute session in horses (*n* = 12) in response to wearing a double bridle and an unfastened crank noseband (UN), a conventionally fastened noseband (CAUN), a noseband fastened with half the conventional area underneath (HCAUN), and noseband fastened with no area underneath (NAUN).

Swallowing rates during treatment showed a stepwise decline as the noseband tightness increased (see Table 6). Swallowing increased for the treatment session compared to the baseline session in the CAUN treatment group (*P* = 0.002). Swallowing increased during the recovery session compared to the treatment session for the HCAUN treatment (*P* < 0.001) and NAUN treatment (*P* < 0.001). Swallowing significantly increased in the recovery compared to baseline sessions for treatment UN (*P* = 0.037), CAUN (*P* < 0.001), HCAUN (*P* = 0.004), and NAUN (*P* = 0.003), indicating a rebound effect (see Fig 6, Table 6).

**Table 6:** Swallowing.

	Treatment			
Session	UN	CAUN	HCAUN	NAUN
	Back-transformed means			
Baseline	1.81	0.79	1.97	1.42
Treatment	2.60	2.67	1.18	0.71
Recovery	3.22	3.70	4.17	3.46
	ratio (s.e.d on log-ratio scale)			
Treatment / Baseline	1.43 (0.2836)	3.4 (0.3756)	0.6 (0.341)	0.5 (0.4262)
Treatment / Recovery	0.8 (0.2442)	0.72 (0.2351)	0.28 (0.3053)	0.2 (0.382)
Recovery / Baseline	1.78 (0.272)	4.7 (0.3636)	2.12 (0.2533)	2.44 (0.2921)
	P value			
Treatment / Baseline	0.211	**0.002**	0.138	0.107
Treatment / Recovery	0.363	0.166	**< 0.001**	**< 0.001**
Recovery / Baseline	**0.037**	**< 0.001**	**0.004**	**0.003**

*P* values for comparisons of swallowing among the four treatments, with averaged back transformed means and ratios in horses (*n* = 12) in response to wearing a double bridle and an unfastened crank noseband (UN), a conventionally fastened noseband (CAUN), a noseband fastened with half the conventional area underneath (HCAUN), and noseband fastened with no area underneath (NAUN).

**Fig 7:**
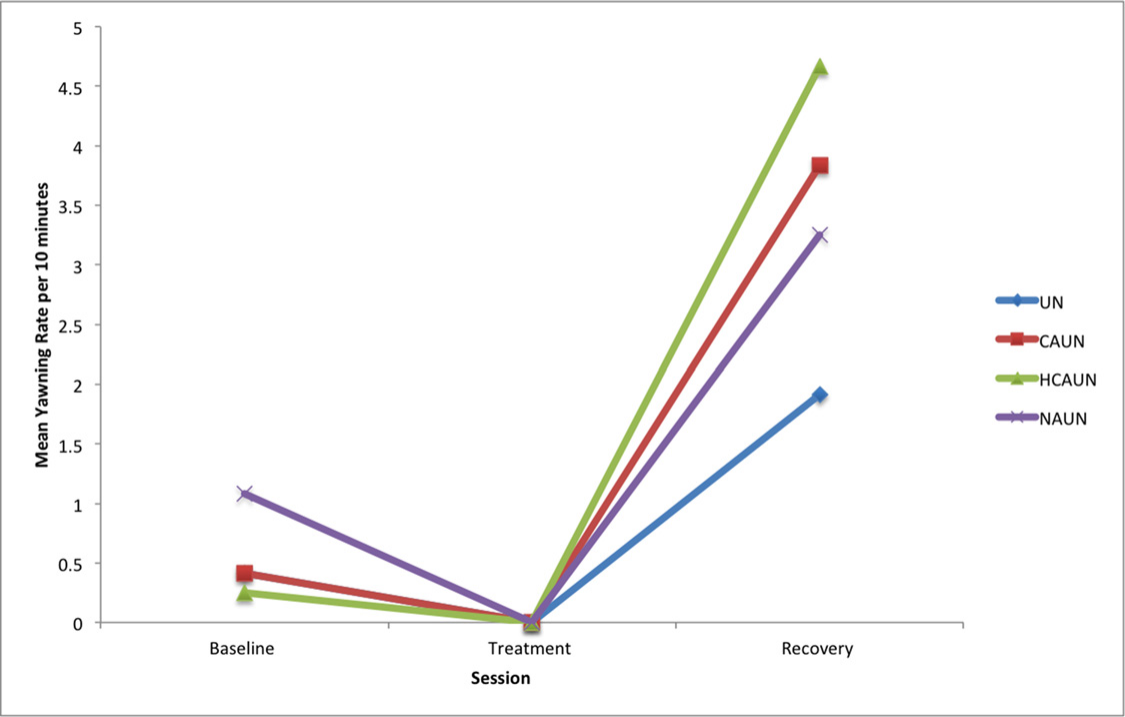
Mean yawning rates per ten-minute session in horses (*n* = 12) in response to wearing a double bridle and an unfastened crank noseband (UN), a conventionally fastened noseband (CAUN), a noseband fastened with half the conventional area underneath (HCAUN), and noseband fastened with no area underneath (NAUN).

In contrast to the baseline and recovery, the frequency of yawning during all treatments was negligible. There was a significant increase in yawning between the baseline and the recovery session for all treatments (UN: *P =* 0.028; CAUN: *P* = 0.001; HCAUN: *P* < 0.001 and NAUN: *P* = 0.015), again indicating a post-inhibitory rebound (see Fig 7, Table 7).

**Table 7:** Yawning.

	Treatment			
Session	UN	CAUN	HCAUN	NAUN
	Back-transformed means			
Baseline	0.42	0.42	0.25	1.08
Treatment	0.00	0.00	0.00	0.00
Recovery	1.92	3.83	4.66	3.25
	ratio (s.e.d on log-ratio scale)			
Treatment / Baseline	N/A	N/A	N/A	N/A
Treatment / Recovery	N/A	N/A	N/A	N/A
Recovery / Baseline	4.6 (0.684)	9.2 (0.653)	18.65 (0.822)	3 (0.444)
	P value			
Treatment / Baseline	N/A	N/A	N/A	N/A
Treatment / Recovery	N/A	N/A	N/A	N/A
Recovery / Baseline	**0.028**	**0.001**	**< 0.001**	**0.015**

*P* values for comparisons of yawning among the four treatments, with averaged back transformed means and ratios in horses (*n* = 12) in response to wearing a double bridle and an unfastened crank noseband (UN), a conventionally fastened noseband (CAUN), a noseband fastened with half the conventional area underneath (HCAUN), and noseband fastened with no area underneath (NAUN).

**Fig 8:**
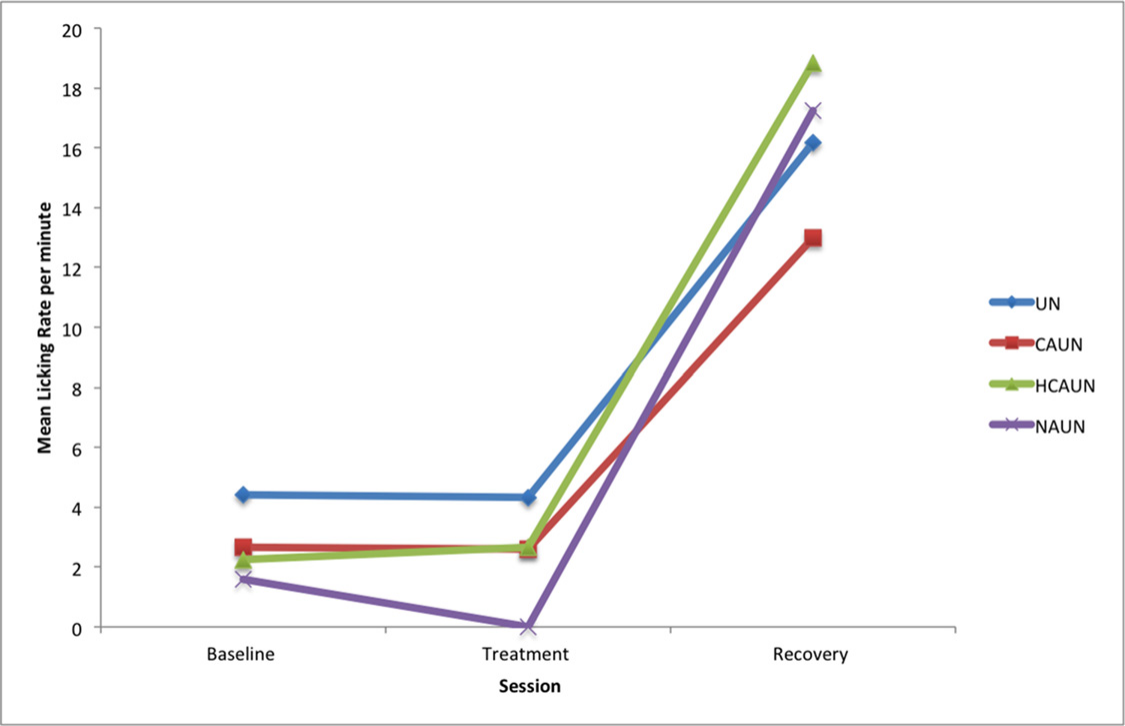
Mean licking rates per ten-minute session in horses (*n* = 12) in response to wearing a double bridle and an unfastened crank noseband (UN), a conventionally fastened noseband (CAUN), a noseband fastened with half the conventional area underneath (HCAUN), and noseband fastened with no area underneath (NAUN).

Licking was eliminated during the NAUN treatment. Licking significantly increased in the recovery session compared to the baseline sessions in all treatments (UN: *P* < 0.001; CAUN: *P* < 0.001; HCAUN: *P* < 0.001 and NAUN: *P* < 0.001, see Fig 8, Table 8). These results represent a post-inhibitory rebound. Significant differences between treatment and recovery were observed for UN (*P* < 0.001), CAUN (*P* < 0.001) and HCAUN (*P* < 0.001).

**Table 8:** Licking.

	Treatment			
Session	UN	CAUN	HCAUN	NAUN
	Back-transformed means			
Baseline	4.41	2.67	2.25	1.58
Treatment	4.33	2.58	2.67	0.00
Recovery	16.17	13.00	18.84	17.25
	ratio (s.e.d on log-ratio scale)			
Treatment / Baseline	0.98 (0.35)	0.97 (0.45)	1.19 (0.47)	N/A
Treatment / Recovery	0.27 (0.28)	0.20 (0.35)	0.14 (0.34)	N/A
Recovery / Baseline	3.66 (0.28)	4.87 (0.35)	8.37 (0.36)	10.89 (0.43)
	P value			
Treatment / Baseline	0.954	0.946	0.710	N/A
Treatment / Recovery	**< 0.001**	**< 0.001**	**< 0.001**	N/A
Recovery / Baseline	**< 0.001**	**< 0.001**	**< 0.001**	**< 0.001**

Comparisons of licking among the four treatments, with averaged back transformed means and ratios in horses (*n* = 12) in response to wearing a double bridle and an unfastened crank noseband (UN), a conventionally fastened noseband (CAUN), a noseband fastened with half the conventional area underneath (HCAUN), and noseband fastened with no area underneath (NAUN).

**Fig 9:**
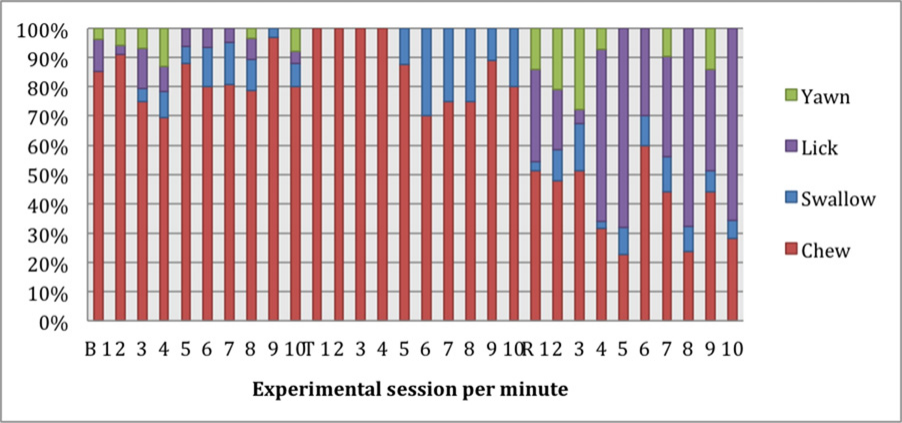
Proportion of all oral behaviors performed during the very tight noseband (NAUN) treatment. B = baseline, T = treatment, R = recovery.

It was expected that at least some oral behaviors might be mutually exclusive, and thus inter-related. Fig 9 shows the distribution of oral behaviors individually as a proportion of all oral behaviors performed before, during and after the NAUN treatment. The relative contribution of licking appears to increase in the recovery period.

There were no significant interactions between treatments and sessions for blinking, ear movements (ears front, neutral and back), or head movements.

## Discussion

The results of the current study show that naïve horses, wearing a noseband with no area available underneath it, demonstrated an increased heart rate, decreased HRV and increased eye temperature compared to when wearing a noseband fitted with half, or more, the conventional recommended area underneath. Horses were stationary during data collection, so cardiac and eye temperature responses recorded could not be attributed to physical activity. The cardiac responses in this study are consistent with previous studies of equine responses to stressful events, such as exposure to novel objects [13, 14]. Increased heart rate, as seen in this study, is considered a physiological indicator of stress. Horses in the current study showed an increase in heart rate when wearing a bridle with no area available under the noseband, suggesting that equipment fitted in this way imposes enough discomfort to provoke a stress response. This stress response may be a result of either the inhibition of normal behaviors or from pain [15] or discomfort [16], or a combination of the two.

It has been suggested that, when compared with heart rate, HRV could be a more detailed and accurate determinant of regulatory functions of the autonomic nervous system in response to stress and that HRV reflects the oscillatory antagonistic influence of the sympathetic and parasympathetic branch of the autonomic nervous system on the sinus node of the heart [17]. The decrease in HRV in the NAUN treatment suggests a stress response but it would be unwise to denounce the practice of binding the jaws together on the basis of HRV responses *in isolation* because the use of HRV to measure stress response is still in its infancy [18].

Eye temperature peaked when the noseband was at its tightest, also suggesting a physiological stress response, as previously reported by McGreevy and others [6]. The scale of the increase in eye temperature reported here is consistent with events such as jugular catheterisation of dairy cows [19], and horses subjected to the acute stress effects of show-jumping [20]. That said, the eye temperature data did not correlate with heart rate or HRV, as might be anticipated. Nevertheless, the increase in eye temperature for NAUN treatment was accompanied by a decrease in HRV and increase in HR. So, it may be that eye temperature and cardiac responses do not arise at exactly the same time. Previous equine research has found significant correlations between eye temperature and salivary cortisol concentrations after a stressful procedure, such as fearful encounters [21].

All oral behaviors were performed in the baseline session, and are thus considered normal behaviors in these horses when wearing a double bridle. Swallowing rates during treatment showed a stepwise decline as the noseband tightness increased. During the tight noseband treatment, the incidence of chewing significantly decreased, relative to baseline, and licking and yawning were eliminated completely. This shows that tight nosebands have the capacity to inhibit normal oral behaviors of horses, as mandibular movement is restricted. Indeed, this is arguably one of the chief purposes of tight nosebands, at least in competitions where penalties accrue if horses open their mouths. It is likely that horses wearing a restrictive noseband have difficulty physically performing normal oral comfort behaviors. Some horses in this study could still chew in even the NAUN treatment, but the frequency of this behavior under these conditions was much reduced. Chewing may have been painful and/or difficult and thus was performed less frequently than in the baseline condition. A reduction in chewing frequency was also seen in the UN treatment, indicating noseband tightness might not be the only factor affecting this behavior. However, during this treatment, there was no significant difference between the treatment and recovery sessions, which may indicate, that once the horse becomes accustomed to wearing the two bits, chewing remains relatively constant when unimpeded by a tightened noseband. It is worth noting that after the UN treatment horses chewed less than during baseline. This may indicate that they have habituated to the bits over the preceding 20 minutes.

The taper gauge used to assess the level of noseband tightness could not fit under the noseband at the nasal planum in the NAUN treatment. However, it is possible that the noseband could have been tightened still further to exclude chewing and other oral behaviors entirely.

Post-inhibitory rebound is shown where the frequency of the behavior increases to higher than the baseline frequency of that behavior after a period of inhibition. It is worth noting that post-inhibitory rebound is the correct term for the phenomenon we are reporting here. However, we accept that inhibition may not be absolute. For example, the reduction in chewing seen in UN treatment may reflect a disinclination to chew as a result of the presence of the bits rather than an absolute inhibition of the behavior. In the current study, following the removal of the bridle, a post-inhibitory rebound was observed for yawning, licking and swallowing after all treatments involving a fastened noseband. Post-inhibitory rebound is a response to the inability to express natural behaviors [8] that indicates an apparent build-up in motivation to express these behaviors. This suggests a state of deprivation [9] which may signal compromised welfare. This study is the first to show a link between post-inhibitory rebound after a treatment and physiological indicators of a stress response during that treatment. Notably, a post-inhibitory rebound occurred in the NAUN treatment in the wake of increased heart rate and eye temperature and decreased HRV. However, post-inhibitory rebounds for swallowing, licking and yawning also occurred after all treatments and were not associated with cardiac indicators of a stress response. These results could indicate that all fastened nosebands restrict oral comfort behaviors, and that the stress response in the NAUN treatment may be more indicative of pain or acute discomfort rather than behavioral restriction alone. A post-inhibitory rebound for each of the oral behaviors, apart from chewing, in the UN treatment, may reflect the horse’s response to the novelty of having to accommodate two bits.

The absence of evidence of a physiological stress response during any given treatment should not devalue post-inhibitory rebound as a behavioral indicator of compromised welfare. The baseline incidence of oral behaviors shows that they may be considered natural behaviors in horses, and the build-up in motivation to perform them that manifests as a post-inhibitory rebound suggests they are likely to perform some function for the horse. As such, physically preventing horses from engaging in these behaviors to the extent where they display a build-up in motivation to perform them must be considered a violation of the freedom to express normal behavior, which is one of the Five Freedoms that are a cornerstone of animal welfare assessment [22].

Nosebands exert pressure values varying between 200 to 400 mmHg around the nasal planum [7]. Such pressures can cause nerve damage in humans, suggesting they could also be detrimental to horse welfare [7]. This prospect is supported by the cardiac results, reported here in the NAUN treatment, that were unparalleled in treatments with looser nosebands. The use of nosebands that restrict natural behaviors and also create a measurable stress response may violate the FEI rule that nosebands are “never as tightly fixed so as to harm the horse” (3). In the light of the current results, the horse sport administrators may need to decide which oral behaviors they can afford to see eliminated in the name of sport.

Compared to baseline and recovery sessions, all treatments virtually eliminated yawning. Post-inhibitory rebound for yawning was observed in all treatment levels. Yawning is of particular interest to equine welfare scientists, because it seems to be triggered by mild distress and negative emotional states [23]. It seems plausible that yawning may represent a coping mechanism to alleviate stress [23] or pressure from the bridle. Yawning in humans has been found to be associated with transitions in arousal level [24] and is thought to be a thermoregulatory mechanism that cools the brain after periods of stress [25]. In budgerigars, handling stress inhibits yawning initially prior to an eventual increase [26]. Miller et al [26] also reported that the budgerigars with higher skin temperatures (namely those that had been most distressed), yawned sooner than others, which may also be reflected in the results of the current study (Fig 9). Horses in this experiment were not physically active or required to engage in cognitively demanding activities whilst undergoing observation. So, the observed increase in yawning following the removal of the noseband and double bridle suggests that yawning may not be simply a sign of physical fatigue. Instead, yawning may be associated with a general de-escalation in arousal [24] as equipment is removed, signalling an end to equipment-dependent activities. Such de-escalation could reflect reduced arousal, conflict and anxiety.

### Limitations

The current study was designed to provide results that are generalizable to large numbers of competition horses and that is why we selected the commonly used crank noseband and the standard taper gauge. However, all horses in this study were naïve to the effects of a crank noseband and double bridle. They were responding not only to the effects of noseband tightening but also to two bits, rather than simply one. This means that further studies, perhaps using the same experimental design, but with one bit only or no bit at all, are justified. It is possible that the combination of a tight crank noseband and two bits virtually immobilises the tongue. This may explain why the rate of swallowing halved, chewing was significantly reduced and licking was absent during this treatment. Future research should investigate both the use of a single bit and crank noseband together with the effects of applying rein tension.

Naïve horses were selected to enable the observation of behavioral and physiological responses that were unaffected by habituation, which may have been quite variable had elite dressage horses, that had been exposed to this treatment, been recruited instead. Future research should quantify long-term exposure to tight nosebands to determine the effect of habituation over time in horses. This would be useful in determining the merit of behavioral observations to assess animal welfare as well as how long horses may be in distress before they habituate to tight nosebands, and the extent to which they habituate.

We acknowledge that those scoring the videos could not be blinded to the treatments. We also acknowledge that scoring space under nosebands relies on operator accuracy and may also be achieved using a Vernier gauge (rounded to the nearest millimetre) or even a simple ruler. Future research in this field could also attempt to calibrate the tightness of extremely tight nosebands. This may be achieved by using tension gauges, such as proposed by Casey et al [7], which could allow researchers to titrate the effects of tight nosebands to determine the tension at which noseband tightness provokes a stress response in horses. It would also be helpful if further research could include longer observations of horses after removal of the nosebands because the interplay between various oral behaviors and cardiac responses in this period may reveal more about importance of each behavior relative to the others.

The current absence of correlations between eye temperature and cardiac measurements may warrant further investigation. Thermography is potentially a valuable tool in animal welfare science because it presents the opportunity to collect physiological data non-invasively. We note that the eye temperature in the current study seemed to normalise quicker during recovery than the heart rate and HRV. This may imply that it is a more sensitive measure than cardiac responses alone. As interest in this prospect grows, a detailed understanding of its potential and the limitations to its use is necessary.

## Conclusions

The current study produced evidence that horses undergo a physiological stress response when wearing a tight noseband in combination with a double bridle. Significant shifts were seen in heart rate, HRV, and eye temperature in association with tight noseband use, suggesting that horses experience pain or discomfort when nosebands are tightened such that there is no space available underneath them. Yawning, licking and chewing were virtually absent and the frequency of swallowing was halved when the nosebands were tightest. Then, yawning, swallowing and licking significantly increased compared to baseline frequencies following the removal of the nosebands and double bridle, indicating a post-inhibitory rebound response. We predict that the impact on horses would increase still further with the addition of rein tension and a rider. The current data indicate that nosebands tightened to the extent that there is no area available underneath them cause a stress response and prevent the expression of normal behavior. To that end, gear stewards in a competition environment should be required to check that each rider is complying with rules that prevent excessive tightening of the noseband. The dressage rules that call for “submission” in horses, demonstrated by a willing acceptance of the bit cannot be properly upheld if the equipment in use prevents the expression of the very behaviors that would indicate oral discomfort and a lack of submission. Further research should focus on the process of habituation to these devices, the measurement of tension in nosebands and the tension level at which the noseband produces a stress response in horses. This may help dissect the sequential roles that inhibition and discomfort play in the emergence of the stress response associated with this practice. Either way, on ethical grounds, the use of relentless pressure to eliminate oral behaviors in pursuit of a competitive advantage may be difficult to justify.
